# The Electronic Properties of O-Doped Pure and Sulfur Vacancy-Defect Monolayer WS_2_: A First-Principles Study

**DOI:** 10.3390/ma11020218

**Published:** 2018-01-31

**Authors:** Weidong Wang, Liwen Bai, Chenguang Yang, Kangqi Fan, Yong Xie, Minglin Li

**Affiliations:** 1 School of Mechano-Electronic Engineering, Xidian University, Xi’an 710071, China; lwbaixdu@foxmail.com (L.B.); cgyangxdu@foxmail.com (C.Y.); kqfan@mail.xidian.edu.cn (K.F.); 2 Department of Mechanical Engineering, Northwestern University, Evanston, IL 60208, USA; 3 School of Advanced Materials and Nanotechnology, Xidian University, Xi’an 710126, China; yxie@xidian.edu.cn; 4 School of Mechanical Engineering and Automation, Fuzhou University, Fuzhou 350108, China

**Keywords:** monolayer WS_2_, electronic properties, O-doped, sulfur vacancy-defect, first-principles study, band gap

## Abstract

Based on the density functional theory (DFT), the electronic properties of O-doped pure and sulfur vacancy-defect monolayer WS_2_ are investigated by using the first-principles method. For the O-doped pure monolayer WS_2_, four sizes (2 × 2 × 1, 3 × 3 × 1, 4 × 4 × 1 and 5 × 5 × 1) of supercell are discussed to probe the effects of O doping concentration on the electronic structure. For the 2 × 2 × 1 supercell with 12.5% O doping concentration, the band gap of O-doped pure WS_2_ is reduced by 8.9% displaying an indirect band gap. The band gaps in 3 × 3 × 1 and 4 × 4 × 1 supercells are both opened to some extent, respectively, for 5.55% and 3.13% O doping concentrations, while the band gap in 5 × 5 × 1 supercell with 2.0% O doping concentration is quite close to that of the pure monolayer WS_2_. Then, two typical point defects, including sulfur single-vacancy (V_S_) and sulfur divacancy (V_2S_), are introduced to probe the influences of O doping on the electronic properties of WS_2_ monolayers. The observations from DFT calculations show that O doping can broaden the band gap of monolayer WS_2_ with V_S_ defect to a certain degree, but weaken the band gap of monolayer WS_2_ with V_2S_ defect. Doping O element into either pure or sulfur vacancy-defect monolayer WS_2_ cannot change their band gaps significantly, however, it still can be regarded as a potential method to slightly tune the electronic properties of monolayer WS_2_.

## 1. Introduction

Recently, two-dimensional (2D) transition-metal dichalcogenides (TMD), such as MoS_2_, WS_2_ and others, have been widely studied because of their excellent properties in mechanics, electronics, optics and so on. As a typical 2D TMD material, WS_2_ has obtained much attention for its extensive application prospects in electronics. Bulk WS_2_ is an indirect gap semiconductor with a band gap about 1.3 eV, while monolayer WS_2_ is a direct gap with a band gap of about 2.0 eV [1]. Pure monolayer WS_2_ has a typical layered structure and consists of a S–W–S sandwich structure. Due to its weak van der Waals interactions between the neighboring WS_2_ layers [2,3], monolayer WS_2_ can be exfoliated from the bulk material. According to available experimental and theoretical observations [4,5,6,7], monolayer WS_2_ exhibits better mechanical, electrical, optical and chemical properties than bulk WS_2_, which can be attributed to the quantum size effect. Therefore, monolayer WS_2_ has many potential applications, for instance, field-effect transistors (FET) [8], photochemical catalysis [9], solid lubricants [10], biosensor device [4] and so on.

In general, doping is an effective method to tune the electric properties of semiconductor materials by selecting the impurity species and adjusting the doping level. There are some available reports about metal and nonmetal element doping monolayer WS_2_ and some other studies about WS_2_. Zhao et al. [11] studied the electronic and magnetic properties of X-doped (X = Ni, Pd, and Pt) WS_2_ monolayers using first-principles calculations based on DFT. Their studies demonstrated that WS_2_ monolayers doped by Ni, Pd, and Pt are ferromagnetic and suitable for thin dilute magnetic semiconductors. Chanana et al. [12] studied the chlorine-doped WS_2_-metal interface and found that WS_2_ physiosorbed with Au has an n-type Schottky barrier height (SBH) while the one chemisorbed with Pd has a p-type SBH. The electronic and optical properties of a vacancy-doped WS_2_ monolayer has been studied by Wei et al. [13]. Their results show that the atomic vacancies give rise to spin polarization around the corresponding and the spin polarization of single W atomic vacancies has a larger range than that for one or two S atomic vacancies.

There are many references related to metal-element-doped WS_2_ monolayers but fewer studies regarding nonmetals are available in the literature, especially those regarding oxygen-doped WS_2_ monolayers. Chen et al. [14] reported an oxygen-assisted chemical vapor deposition method to grow the monolayer MoS_2_ and found that introducing a small amount of oxygen can effectively improve the growing region of MoS_2_. WS_2_ and MoS_2_ belong to TMDs, therefore, we want to probe the influence of oxygen element and the cognate element of sulfur, on pure WS_2_ monolayers, especially in electronic structure. The monolayer WS_2_ can be prepared through different routes, these being mainly mechanical and chemical exfoliation [15,16]. Gutiérrez et al. [17] reported the synthesis of single triangular microplatelets of WS_2_ via the sulfurization of ultrathin WO_3_ films. In general, the point-defects are created in the process of synthesizing and transferring and three types of vacancy defects, such as sulfur (S) single-vacancy, tungsten (W) single-vacancy and sulfur (S) di-vacancy, which have been proven [18,19].

In the present study, we focus on the influences of oxygen (O) element on the electronic properties of both pure monolayer WS_2_ and two types of sulfur vacancy-defects monolayer WS_2_, including S single-vacancy and S di-vacancy that are mentioned above. The electronic properties of O-doped pure and sulfur vacancy-defect WS_2_ monolayers are studied using first-principles calculations based on the density functional theory (DFT). The observations from our DFT calculations show that the doping concentration has some effect on the band gap of monolayer WS_2_. The relatively low doping concentration can broaden the band gap compared with pure monolayer WS_2_, while the higher doping concentration presents an opposite trend. It is also found that O-doped sulfur single-vacancy (V_1S_) WS_2_ can broaden the band gap, while O-doped sulfur di-vacancy (V_2S_) can weaken the band gap to some extent. The present study predicts O-doped monolayer WS_2_ to be a potential channel to slightly changing its electronic properties, which may provide some help for future applications of WS_2_ semiconductor devices.

## 2. Physical Modeling and Simulation Methods

**Figure 1:**
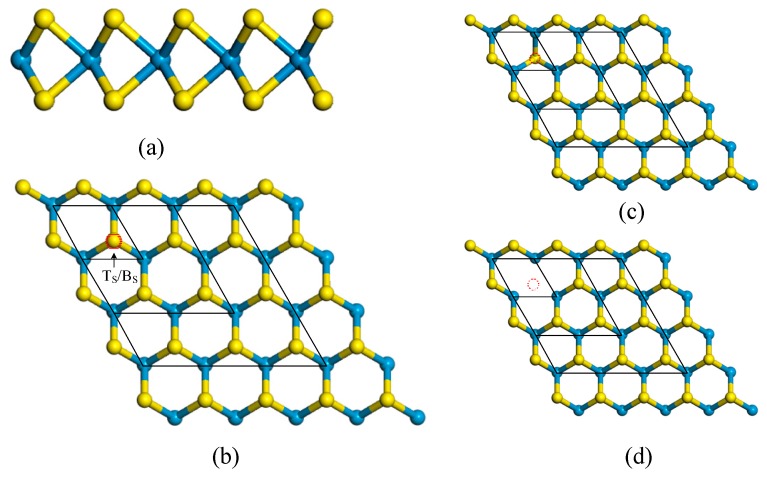
Schematic structure of pure 5 × 5 × 1 monolayer WS_2_: (**a**) side view; (**b**) top view; and supercell models of the defective monolayer WS_2_: (**c**) V_S_ and (**d**) V_2S_. T_S_ represents the S atom on the top site, and Bs represents the S atom on the bottom site. The red solid circle represents the doping position of O atom in 2 × 2 × 1, 3 × 3 × 1, 4 × 4 × 1 and 5 × 5 × 1 WS_2_ supercell in (**b**). The red dotted circles given in (**c**,**d**) represent the positions of the vacancy of 2 × 2 × 1, 3 × 3 × 1, 4 × 4 × 1 and 5 × 5 × 1 WS_2_ supercell. The yellow and blue balls represent S and W atoms, respectively. In (**b**–**d**), the black solid lines represent the computational model of 2 × 2 × 1, 3 × 3 × 1, and 4 × 4 × 1 WS_2_ supercell.

Monolayer WS_2_ is a direct band gap semiconductor, with a band gap of about 2.0 eV [1]. The layered WS_2_ consists of stacked S–W–S and has a *P*6_3_/*mmc* space group symmetry with the W atoms having a trigonal prismatic coordination with the S atoms as shown in Figure 1a. In the present study, an oxygen (O) atom is used to substitute a sulfur (S) atom of pure and sulfur vacancy-defect monolayer WS_2_ in order to investigate the effect of O element on the electronic properties of monolayer WS_2_. To study the effect of doping concentration on electronic properties of pure monolayer WS_2_, one S atom was substituted by one O atom in 2 × 2 × 1, 3 × 3 × 1, 4 × 4 × 1 and 5 × 5 × 1 monolayer WS_2_ supercell, respectively. Moreover, in order to probe the influences of O element on the electronic properties of sulfur vacancy-defect monolayer WS_2_, two possible vacancy-defect types, including S single-vacancy (V_S_) shown in Figure 1c, and S di-vacancy (V_2S_) shown in Figure 1d, are discussed in the present study. V_S_ is obtained by removing T_S_ (the S atom on the top site) and V_2S_ is obtained by removing the two S atoms both T_S_ and B_S_ (the S atom on the bottom site). To investigate the influence of O element on the electric properties, one O (1-O) atom is introduced in these two types of sulfur vacancy-defect WS_2_ from a 2 × 2 × 1 to 5 × 5 × 1 monolayer WS_2_ supercell, and DFT calculations are carried out in the following studies.

Our numerical calculations are performed using the first-principles method based on DFT. All the optimizations of geometry and calculations of electronic properties are performed within the Vienna ab initio simulation package (VASP) based on the projected augmented wave (PAW) method [20,21]. Electron exchange and correlation effects are dealt with in the generalized gradient approximation (GGA) in the Perdew–Burke–Ernzerhof (PBE) parametrization [22]. During all DFT calculations, the cut-off energy for the plane-wave expansion of the wave functions is set as 500 eV. The Brillouin zone (BZ) is obtained by using the Monhkorst–Pack [23] method and the k-points 5 × 5 × 1 is utilized to relax the atomic positions. All structures are fully relaxed until the maximum forces acting on each atom and the total energy are less than 0.01 eV/Å and 10^−6^ eV, respectively. To avoid the interaction between the adjacent monolayers, the vacuum layer along the *z*-direction is set as 20 Å.

## 3. Results and Discussion

### 3.1. Structure Properties

First, by using a 2 × 2 × 1 supercell, the structural parameters of the pure monolayer WS_2_ are obtained through DFT calculations in the present study, as given in Table 1. It also can be found that the distance between W atom and the adjacent S atom and the W–S–W angle between the nearest neighbor S atom are lightly larger than the experiment values, which are attributed to the reason that GGA always overestimates parameters. Then, one sulfur (S) atom is substituted by an oxygen (O) atom and the relaxing results on the structural parameters of O-doped monolayer WS_2_ are shown in Table 1. The lattice parameter of O-doped WS_2_ is *a* = *b* = 3.132 Å which is smaller than that of pure WS_2_. The bond length of the W–O (*d*_W–O_ = 2.084 Å) in the O-doped monolayer WS_2_ is tighter than the W–S bond length (*d*_W–S_ = 2.416 Å) of pure WS_2_, which is caused by the different atomic radius. The S–W–O angle is smaller than the S–W–S angle compared with pure monolayer WS_2_, while the W–O–W angle is larger than the W–S–W angle. All these results indicate that there is a slight distortion as one O atom is doped to the 2 × 2 × 1 monolayer WS_2_ supercell.

### 3.2. Band Structure and Density of States (DOS)

#### 3.2.1. O-Doped Pure WS_2_

**Figure 2:**
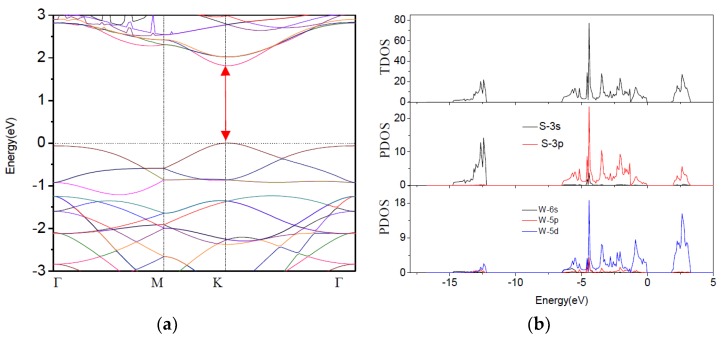
(**a**) Band structure of pure monolayer WS_2_ and (**b**) TDOS and PDOS of pure monolayer WS_2_. The band gap are shown with red arrows.

In order to study the influence of O element on the electronic properties of monolayer WS_2_, we first calculated the band structure of pure monolayer WS_2_ along the high symmetry across the first Brillouin zone, as shown in Figure 2a. The Fermi level is set as the maximum of the valence band, then, the total density of states (TDOS) and partial total density of states (PDOS) for pure WS_2_ are shown in Figure 2b. Apparently, the calculated band gap is 1.79 eV. This is smaller than the previous theoretical value [25], which is due to different choices of plane-waves cutoff and k-point grid. It is very easy to observe that the conduction band minimum (CBM) and the valence band maximum (VBM) are at the same high symmetry K point, which indicates that monolayer WS_2_ is a direct band gap semiconductor that agrees well with the previous calculation. In addition, it can be concluded that the CBM and VBM of pure WS_2_ monolayers are attributes of the W 5*d*- and S 3*p*-states in Figure 2b.

**Figure 3:**
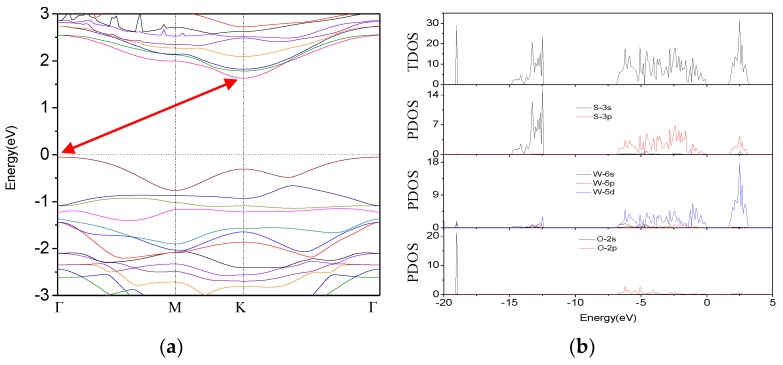
(**a**) Band structure of O-doped monolayer WS_2_ and (**b**) TDOS and PDOS of O-doped monolayer WS_2_. The band gap are shown with red arrows.

As one S atom of pure WS_2_ is substituted by one O atom, i.e., 12.5% O doping, the calculated band structure of 2 × 2 × 1 WS_2_ supercell is given in Figure 3a, and its TDOS and PDOS are shown in Figure 3b. From TDOS and PDOS results, it can be concluded that the contributions in the gap formation of W atom and S atom have little change compared with the pure WS_2_ monolayer. It is obvious that the contribution of the states of O is at a low energy band or close to the Fermi level, that is to say, O doping has no obvious effects on the maximum of the valence band and the minimum of the conduction band from Figure 3b. Nevertheless, in the band structure given in Figure 3a, it is noticed that the CBM is at K point and the VBM is at Γ point, which indicate that the direct band gap transforms into an indirect band gap after one O atom was doped to the 2 × 2 × 1 WS_2_ supercell. We can also obtain that the band gap becomes 1.630 eV which is reduced by 8.9% compared with pure monolayer WS_2_. In short, O doping has some influences on the band gap of monolayer WS_2_. In the following section, other different O doping concentrations will be investigated to probe their influences on the band gap.

**Figure 4:**
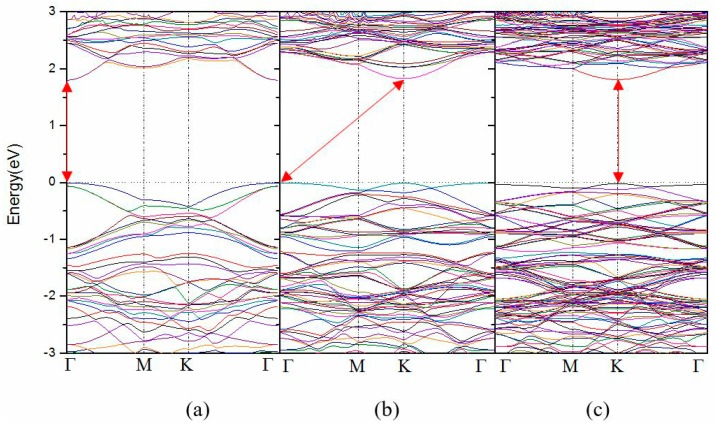
Band structure of different sizes of monolayer WS_2_ supercell with one substitutional O atom: (**a**) 3 × 3 × 1; (**b**) 4 × 4 × 1; and (**c**) 5 × 5 × 1. The band gaps are shown with red arrows.

Figure 4 displays the band structure for one substitutional O atom in a 3 × 3 × 1, 4 × 4 × 1 and 5 × 5 × 1 supercell of monolayer WS_2_, i.e., 5.55%, 3.13% and 2.00% O doping concentration, respectively. As shown in Figure 4a, the band extrema are both locate at a Γ-point which indicates a direct band gap of 1.800 eV, which is slightly opened compared with the pure WS_2_. For the case of 4 × 4 × 1 supercell in Figure 4b, the calculated band gap, 1.820 eV, is further broadened with the VBM at the Γ-point and the CBM at K-point which displays an indirect band gap. The band gap of 5 × 5 × 1 supercell is 1.785 eV with the band extrema at K-point in Figure 4c, which indicates a direct band gap, and it should be noticed that the band gap, 1.785 eV, is close to the pure value which we think is caused by the very low doping concentration. The alternate transformation characteristics of the band gap which can be found in Table 2 are the results of the band structure folding due to the symmetry of the hexagonal cell of WS_2_. In summary, it can be found that the relatively high doping concentration of 12.50% can reduce the band gap and the relatively low doping concentrations of 5.55% and 3.13% can open the band gap to a certain degree, while the very low doping concentration of 2.00% has little effect. In the case of O-doped pure WS_2_, the observations show that O element has some effects on the band gap of monolayer WS_2_ in a small range.

#### 3.2.2. O-Doped Sulfur Vacancy-Defect WS_2_

**Figure 5:**
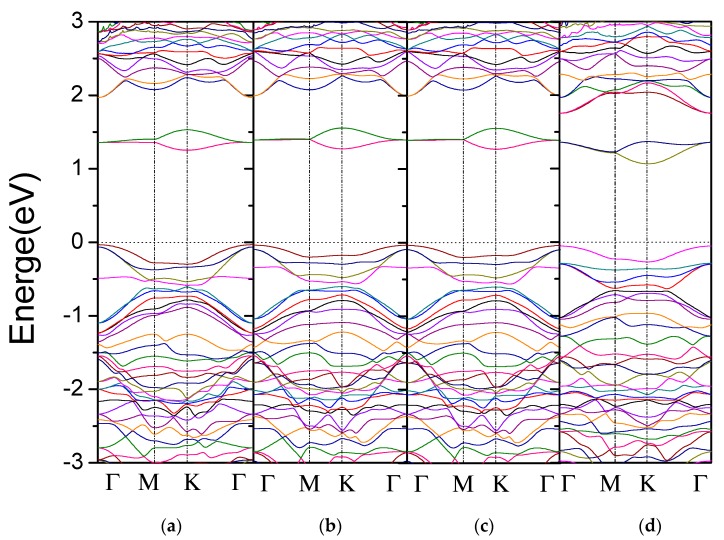
Band structure of a 3 × 3 × 1 WS_2_ monolayer with (**a**) V_S_; (**b**) V_S_-O; (**c**) V_2S_ and (**d**) V_2S_-O. The V_X_-O (X = S, 2S) means 1-O atom doped WS_2_ monolayer with V_X_ types of vacancy defects.

Next, we focus on the effect of O element on the electronic properties of sulfur vacancy-defect monolayer WS_2_. First, we calculated the band structures of both the monolayer WS_2_ with V_S_ defect shown in Figure 5a and the monolayer WS_2_ with V_2S_ defect shown in Figure 5c. Compared with the pure case in Figure 2a, these two defective cases introduce impurity bands inside the band gap due to the atomic vacancies, which are consistent with the studies of Zhao et al. [26] and Krivosheeva [27]. The other sizes of supercells give the same results, so we only display the band structure of 3 × 3 × 1 WS_2_ monolayer in this paper. At the same time, we also display the band structures after 1-O atom doping of the monolayer WS_2_, respectively with V_S_ and V_2S_ in Figure 5b,d. From Figure 5 and Table 3, one can find that the WS_2_ monolayers with V_S_ and V_2S_ have a band gap of 1.250 eV and 1.200 eV, respectively. After introducing 1-O atom into these two defective WS_2_, their band gaps become 1.270 eV and 1.060 eV, respectively. For the case of V_2S_, its band gap is obviously reduced, while for the case of V_S_, its band gap is slightly opened; similar results can be observed for other supercells from Table 3. The larger supercell is, the smaller the band gap changes, in other words, with decreasing of O doping concentration, the band gap is hardly changed. Therefore, we can conclude that if the supercell is larger than 5 × 5 × 1, the doping of 1-O atom in WS_2_ monolayers with V_S_ and V_2S_ can be neglected.

**Figure 6:**
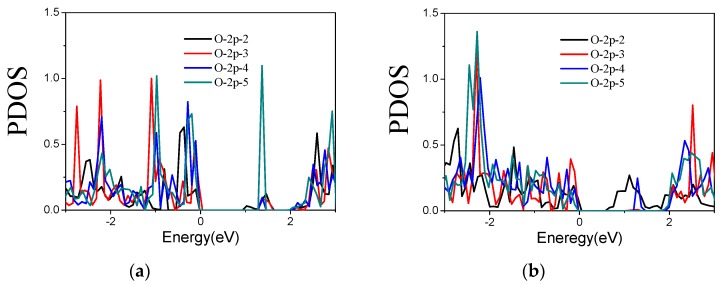
PDOS of 1-O-doped WS_2_ monolayers with (**a**) V_S_ and (**b**) V_2S_ types of vacancy defects. The value of O-2p-X (X = 2, 3, 4 and 5) represents the size of WS2 monolayer supercell (X × X × 1).

In order to further probe the effect of O atom on sulfur vacancy-defect WS_2_ monolayers, we calculate the PDOS of 1-O doped two defective WS_2_ monolayers with supercell sizes from 2 × 2 × 1 to 5 × 5 × 1. The contribution in the gap formation given by *s*-states of O atom is far away from the Femi energy level, but that given by *p*-states is near the Femi energy level, which are similar to those shown in Figure 3b. Herein, only the PDOS of *p*-states of O atom are given in Figure 6. Compared with the band gaps given in Table 3, it is obvious that the main contribution in band gap formation is given by *p*-states of O atoms. It is also found that for these two defective cases, the first non-zero positive value of PDOS in the conduction band presents at a higher energy as the supercell size increases; as a consequence, there is a slight contribution of O atom to the bottom of the conduction band, which just describes the reason of the changes of the band gap in Table 3.

## 4. Conclusions

In this paper, DFT calculations are carried out to investigate the electronic properties of O-doped monolayer WS_2_ with 12.5%, 5.55%, 3.13% and 2.00% O doping concentrations by using the first-principles methods. It is found that the band gap is smaller than the pure value, with the direct band gap transforming into an indirect band gap after replacing one S atom by one O atom in a 2 × 2 × 1 monolayer WS_2_ supercell. The calculation results show that the relatively low doping concentrations are helping to open the band gap to some extent, such as 5.55% and 3.13% O concentrations, and the relatively high O doping concentration (12.5%) can reduce the band gap compared with that of pure WS_2_, while the very low doping concentration of 2.00% has little effect on the band gap. Finally, the effects of O element on the electronic properties of monolayer WS_2_ with two types of sulfur vacancy defects including V_S_ and V_2S_ are investigated. The DFT simulation results show that O doping can broaden the band gap of monolayer WS_2_ with V_S_ defects in a small range, but weaken the band gap of monolayer WS_2_ with V_2S_ defects to some extent. In summary, the band gaps of either pure or sulfur vacancy-defect monolayer WS_2_ could not be tuned significantly by doping O element. However, the influence of O doping on band gaps should not be ignored especially for high doping concentrations and O doping can still be regarded as a potential method to slightly tune the electronic properties of monolayer WS_2_.

## Figures and Tables

**Table 1:** Structural parameters of pure and O-doped 2 × 2 × 1 monolayer WS_2_.

Configuration	*a* (Å)	*d*_W–S_ (Å)	*d*_W–O_ (Å)	∠_W–S–W_	∠s–w–s	∠_W–O–W_	∠_S–W–O_
Pure WS_2_ (cal.)	3.181	2.416	-	82.34°	81.04°	-	-
Pure WS_2_ (exp.)	3.153	2.405	-	81.93°	81.60°	-	-
O-doped WS_2_ (cal.)	3.132	-	2.084	-	-	94.02°	75.57°

Notes: “cal.” means the DFT calculation value in present study; “exp.” means the experimental value in a previous study [24].

**Table 2:** The band gap and its type of O-doped pure WS_2_ monolayer with different supercells.

Model	Band Gap	Type
2 × 2 × 1	1.630 eV	indirect
3 × 3 × 1	1.800 eV	direct
4 × 4 × 1	1.820 eV	indirect
5 × 5 × 1	1.785 eV	direct

**Table 3:** The band gap size of a WS_2_ monolayer with V_S_, and V_2S_ types of vacancy and after 1-O doping above two defective cases (denoted by V_S_-O and V_2S_-O; The V_X_-O (X = S, 2S) means 1-O atom doped WS_2_ monolayer with V_X_ types of vacancy defects.) in different size supercells. The unit is eV.

Configuration	2 × 2 × 1	3 × 3 × 1	4 × 4 × 1	5 × 5 × 1
V_S_	0.910	1.250	1.340	1.362
V_S_-O	0.980	1.270	1.352	1.363
V_2S_	0.980	1.200	1.290	1.283
V_2S_-O	0.840	1.060	1.280	1.277
